# Development and evaluation of a quality improvement educational video on joint contractures for care home staff

**DOI:** 10.1136/bmjoq-2024-002923

**Published:** 2024-12-27

**Authors:** Hina Tariq, Joel Dunn, Samantha Forrester, Kathryn Collins, Sam Porter

**Affiliations:** 1 Faculty of Health and Social Sciences, Bournemouth University, Bournemouth, Dorset, UK; 2 Dorset HealthCare NHS Foundation Trust, Poole, Dorset, UK; 3 Bournemouth University, Faculty of Health and Social Sciences, Bournemouth, UK

**Keywords:** Quality improvement, Long-Term Care, Nursing homes, PDSA, Staff Development

## Abstract

**Background:**

Contractures are a debilitating problem for individuals living in long-term care settings. However, there is a lack of education and training among the care staff regarding the identification of risk factors related to contractures and the preventive strategies that can decrease their development or progression. Addressing this knowledge gap has the potential to improve the quality of care provided to residents in care homes.

The objective of this quality improvement (QI) project was to investigate the impact of a newly developed educational video on the awareness, knowledge and understanding of contractures among the care staff.

**Methods:**

This QI project involved two sequential Plan-Do-Study-Act cycles and employed a pre and post-test design to evaluate the impact of the contracture educational video. Primary outcomes were assessed using paper surveys to capture prevideo and postvideo levels of knowledge and understanding of contractures. Furthermore, both verbal and written feedback from participants were gathered to identify areas of strengths and improvement.

**Results:**

Baseline data revealed that about 56% of the care staff lacked knowledge and understanding of contractures with another 33% reporting possessing only basic knowledge. Following the video intervention, percentage of care staff who reported good knowledge and understanding increased to 67% while 22% reported basic knowledge and understanding of contractures. The care staff suggested changes to the video to improve accessibility of the information, this was incorporated in the refilming of the video.

**Conclusion:**

This QI project demonstrated that the introduction of a contracture educational video is a feasible and positively received method of enhancing awareness, knowledge and understanding of contractures among care staff. Educating care staff about the risk factors and prevention strategies for contractures will potentially improve their ability to identify the risk of contractures and help prevent their occurrence, ultimately enhancing the quality of care of the residents.

**WHAT IS ALREADY KNOWN ON THIS TOPIC**

- Contractures are a preventable but common consequence of immobility among individuals living in long-term care settings.
- Contractures are associated with increased dependence and poorer quality of life.
- There is a lack of education and training on contractures among the care staff.

**WHAT THIS STUDY ADDS**

- After watching an educational video care home staff increased their knowledge, understanding and awareness of contractures.
- This is the first-quality improvement project that evaluates the knowledge and understanding of contractures among care staff after watching an awareness video.
- Education and training on the key risk factors and preventative strategies for contractures will equip the care staff with the ability to identify contracture risk, initiate early interventions within the care home setting, escalate timely referrals to the healthcare professionals and consequently improve the quality of care of the residents.

**HOW THIS STUDY MIGHT AFFECT RESEARCH, PRACTICE OR POLICY**

- Improved knowledge, understanding and awareness of contractures can help to prevent contractures.
- More research is needed to identify longer term retention of the educational information and if the new knowledge impacts on care home residents’ the care and development or progression of contractures.
- If a contracture education tool can contribute to earlier intervention and prevention of contracture development or progression, this education can be incorporated into health and social care policy.

## Introduction

Current evidence indicates a high prevalence of joint contractures among residents of long-term care facilities ranging from 20% to 75%,^1^ however, they remain an important healthcare challenge that has not received sufficient attention.^2^

The primary risk factor associated with the onset of contractures is immobility.^3^ A significant proportion of individuals residing in long-term care facilities exhibit sedentary behaviour, which consequently leads to a higher incidence of developing contractures.^4^ A study by Selikson *et al* revealed that 70.5% of non-ambulatory care home residents developed contractures in contrast to the ambulatory group, which did not develop any.^5^

Most direct care to care home residents, including mobility assistance is provided by the care staff.^6^ The nursing staff at these facilities are increasingly becoming reliant on care staff to monitor and report changes in the health and behaviour status of the residents.^6^ Fisher and Ellwood, in their study, reported that physiotherapists, working in a care home setting, have observed poor postural management and limited opportunities for physical activity for the residents under their care.^7^

The initial onset and progression of contractures typically do not cause pain or disability; residents only experience pain in the joints when they attempt to move them beyond the soft-tissue restrictions.^8^ Consequently, both individuals and their caregivers often fail to identify the development of contractures until they significantly interfere with their activities of daily living. Once developed, contractures tend to lead to a cascade of irreversible impairments perpetuating a vicious cycle that further worsens the condition.^3^ This escalation leads to an increased burden of care, difficulty in moving and handling tasks for the carers and increased financial costs for the care home as the needs of the affected individual increase.^3^

For the caregivers to recognise the risk associated with limited mobility and contractures in a timely manner, and to escalate early interventions at the care home level and timely referrals to healthcare professionals if required, it is necessary for them to acquire training on contracture awareness. In addition, the provision of advice and guidance on physical activity and effective positioning to the care staff will help maintain the independence of frail people or those with a disability and improve their quality of life. It would also serve to support caregivers, reduce care costs through efficiency savings and reduce hospital admissions.^7^

This quality improvement (QI) project supported by NHS Dorset aimed to develop an educational video to improve the awareness, knowledge and understanding of contractures among the care staff at local care homes.

## Methods

This report conforms to the Standards for Quality Improvement Reporting Excellence (SQUIRE) 2.0 guidelines.^9^

### Study design and setting

This project adopted QI approach with a pre and post-test design to evaluate a video-based educational intervention for care home staff caring for residents with contractures or at risk of contractures. The project followed the framework outlined by the Institute for Health Care Improvement’s model for improvement,^10^ which consists of two components: three core questions and the Plan-Do-Study-Act (PDSA) cycle which is used to test changes in actual work contexts to see if they lead to improvement.^10^ This in turn informed the three phases of this project: (1) analysis of the problem and development of the interactive training session, (2) implementation of the training session and (3) evaluation of the training session’s impact on care staff knowledge and understanding of contractures.

The project was conducted at two local care homes in Dorset, both of which offer residential, nursing, dementia, palliative/end of life and respite care.

### Participants

The population included the carers who provide or organise the care of the residents with contractures or at risk of developing contractures.

### Patient and public involvement

NHS Dorset conducted an educational needs survey locally with the healthcare professionals focusing specifically on contractures. The survey aimed to identify the current gaps in areas where staff may lack training and educational resources related to the prevention and management of contractures. The findings highlighted poor awareness of contractures among care staff. Additionally, the survey findings indicated that most respondents (73%) favoured bite-sized educational videos as the preferred method for virtual training on contractures.

The educational video in this QI project was developed to increase the knowledge and understanding of risks and prevention of joint contractures among the care home staff. The methods reported here demonstrate engagement with the care staff who are the end-users from baseline through to the conclusion of this project. The initial PDSA cycle involved testing the educational video with care staff who then provided feedback to inform the required changes tailored to their needs. The changes were subsequently incorporated into the refilming of the video before its final dissemination.

### Service improvement team and analysis of the problem

The service improvement team comprised two expert physiotherapists (HT and JD) and one occupational therapist (SF) who was also working as a QI engagement and development facilitator.

The script of the video was mainly developed by HT and JD and was based on previous research evidence gathered by HT, KC, JD, DT, SA and SP.^3 11^

SF and three other volunteers who featured in the video (one nursing practitioner, one rehabilitation assistant and one care staff member) also gave their input during the development of the script. All team members collaborated closely to analyse the problem, design the video intervention, assist with data collection, analyse and generate change ideas.

A fishbone analysis allows the service improvement team to fully understand the nature of the problems, the underlying causes contributing to the problem and as a result producing outcomes, which reflect the solution of the problem.^12^ The service improvement team utilised fishbone analysis to identify and assess the potential causes of minimal or no training for care home staff supporting residents with contractures or at risk of developing contractures (online supplemental material 1). A series of meetings were held among the service improvement team members and with the gatekeepers (senior care staff members) of the care homes to address the solutions to major causes of the problem.

### Strategy and processes

#### PDSA cycle 1

Plan:

- Define the goals of the QI project: using an interactive session and educational video to create awareness and confidence of care home staff in Dorset, when supporting people who have contractures or are at risk of developing contractures.
- Establish-specific learning objectives of the training session.
- Develop video training material using the latest research evidence.
- Ensure that the language of the training material is tailored to the target audience, that is, care home staff.
- Peer review the training material with healthcare professionals with experience of working with residents with contractures.
- Incorporate interactive elements such as animations, pictures and useful acronym to enhance learning retention.
- Engage relevant stakeholders in the planning process to ensure their support and collaboration throughout the project. In this cycle, we collaborated with two care homes, therefore the learning and development managers at both facilities were engaged.

Do:

- Conduct the planned interactive video training sessions at selected care homes (intervention).
- Administer pre and post-training survey to measure the increase in staff knowledge and understanding of contractures.
- Gather qualitative feedback via open-ended questions in the training survey from the care home staff regarding the video content, and overall experience including any areas of confusion and suggestions for improvement.

Study:

- Analyse the results to explore the change in knowledge and understanding of contractures after watching the educational video.
- Review feedback to identify strengths, weaknesses and areas for improvement in the educational video.

Act:

- Incorporate feedback from the care home staff to revise and refine the training material.
- Modify and adjust delivery approach based on the identified areas for improvement.
- Disseminate the educational video to a wider audience on media platforms.

#### Intervention

**Figure 1:**
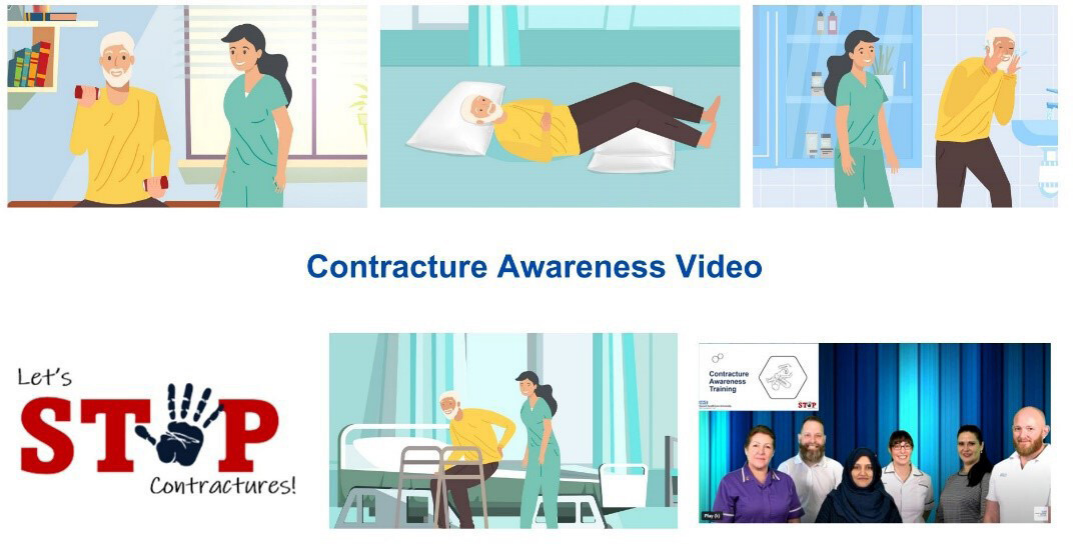
The figure presents selected images from the educational video on contracture awareness for care home staff.

The training session was delivered face-to-face by HT and SF. The training was a 30 minute interactive session, which consisted of a PowerPoint presentation, a contracture awareness video (figure 1) and administration of a paper-based pre and post-survey. The PowerPoint presentation started with Introduction of the trainees and overview of the session, which was then followed by administration of the pre-video survey measuring the baseline knowledge of the care staff. The care staff were asked about their demographics, rate their current knowledge and understanding of contractures on a Likert scale from 0 (no knowledge and understanding) to 3 (extensive knowledge and understanding) and how much they agreed with the statement, *I provide care for residents with contractures* from 0 (never) to 3 (always). They were also asked if they had previously attended any training on contractures and if they ever used a tool/method to assess the risk of contractures in the residents.

#### Training video

The video script was written in plain English and non-technical language to accommodate the care staff with no medical background. The video employed a slide show format and featured two physiotherapists, one occupational therapist, one rehabilitation assistant and one nursing practitioner as trainees, which reflects a multidisciplinary input in contracture prevention and management. The contracture knowledge concepts addressed in the video included a clear description of contractures, levels of severity of contractures, the impact of contractures on the individual, the carers and on the care home, the conditions in which contractures are most prevalent, most common risk factors and how contractures can be prevented in a care home. An acronym, Strength, Treatment, Opportunity to engage, Positioning (STOP) was utilised to describe the prevention strategies that can be utilised by the care staff to prevent contractures. S for strength, T for treatment, O for opportunity to engage and P for positioning (figure 1). The video also added useful links to easy resources for physical activity and strengthening exercises that are accessible by the care homes. Useful animated pictures and videos were incorporated into the video to demonstrate the prevention strategies (figure 1).

#### Post-video survey

A post-video survey was employed to measure the change in knowledge and written and verbal qualitative feedback was gathered to identify areas of strengths and improvement.

### Analysis

Descriptive statistical analyses were used to report on the categorical variables while the qualitative feedback on the training was analysed using thematic analysis.^13^

## Results

### Quantitative results

A total of nine care staff participated in the training. The demographic characteristics of the participants are presented in table 1.

**Table 1:** Demographic characteristics

ID	Job role	Years of experience	Specialty
1	Learning and development facilitator	1–5 years	Elderly care
2	Learning and development manager	5–10 years	Elderly care
3	Healthcare assistant	1–5 years	Elderly care
4	Healthcare assistant	20+ years	Elderly care
5	Clinical support manager	20+ years	Elderly and complex care
6	Healthcare assistant	1–5 years	Dementia care
7	Senior healthcare assistant	1–5 years	Elderly care
8	Well-being assistant	1–5 years	Elderly care
9	Care practitioner	20+ years	End of life care, dementia care, nursing care

#### Pre-video survey

**Figure 2:**
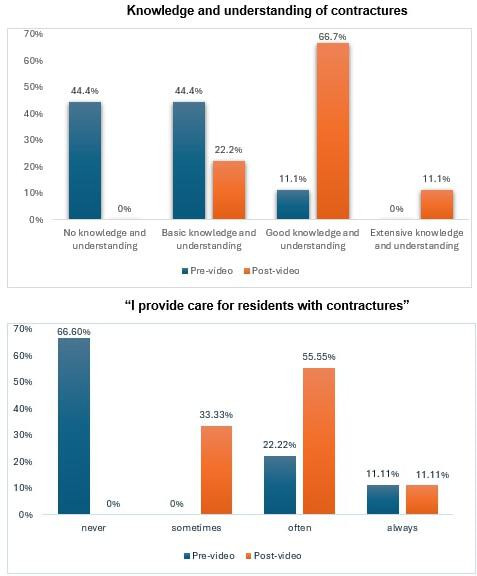
The figure compares pre-video and postvideo survey results on contracture knowledge and understanding among care home staff.

The pre-video survey (figure 2) demonstrated that four out of nine participants had no knowledge and understanding of contractures (44.44%), four had basic knowledge and understanding of contractures (44.44%) and one had good knowledge and understanding of contractures (11.11%). When asked, how much you agree with the following statement: ‘*I provide care for residents with contractures’*, six reported, ‘never (66.66%), two reported, often (22.22%) and one reported, always (11.11%).

When asked *if they ever used a tool or method to assess the risk of contractures for their residents*, eight out of nine respondents, reported, ‘no’ (88.88%) while one reported, ‘yes’ (11.11%) (with the help of the occupational therapist and allocated forms). All nine participants reported that they had never received any training related to contractures or postural management before (100%).

#### Post-video survey

Post-video survey (figure 2) demonstrated that six out of nine participants reported good knowledge and understanding of contractures (66.66%), two reported basic knowledge and understanding of contractures (22.22%) and one reported extensive knowledge and understanding of contractures (11.11%). When asked, how much you agree with the following statement: ‘I provide care for residents with contractures’, five reported ‘often’ (55.55%), three reported ‘sometimes’ (33.33%) and one reported ‘always’ (11.11%).

### Qualitative findings

The following key themes emerged from the qualitative analysis of the feedback provided by the care staff supported by the quotes:

(1) Improved understanding of contractures.

Participants expressed a greater understanding of contractures through the training.

This training has been very beneficial…… this training gave me an understanding of contractures which I didn't have before.

This training session has been very useful and helped me gain an understanding of what contractures are and how to help prevent them from occurring.

(2) Positive feedback on training material

Most participants found the training content useful and informative and appreciated the correlation of important clinical aspects with contractures.

I liked the way this learning cross-references with other core aspects such as pressure ulcer care improving and positioning etc.

I believe the resources mentioned in the learning are also beneficial. Pillows used in care homes are easy to source and other prevention ideas such as squeezy balls.

Participants found the STOP acronym and the vicious cycle of contractures very helpful and informative.

I have more knowledge about: vicious cycle of contractures, STOP—Key to prevent contractures: Strength, Treatment, Opportunity to engage, Positioning.

(3) Engaging presentation and design

Participants found the overall training material engaging and designed to maintain attention.

Well presented and designed in a way to keep attention.

(4) Areas for improvement

Some participants suggested some weaknesses and areas for improvement in the educational video. There were suggestions around the inclusion of pictures related to contractures to enhance better understanding, to increase the size of text in certain visuals (eg, in the vicious circle diagram) for better readability. Moreover, avoiding academic language in some areas and making the script more relatable to the carers to ensure accessibility and relevance to care home staff.

Relate it to carers so that it feels a bit more personal than clinical. Use, you, and your staff/colleagues instead of a third person (care home and their staff)

Clearer messages that support the care staff and they are able to do this without clinical opinion.

In addition, there were suggestions to feature the care home staff in the educational video along with other healthcare professionals to provide a more authentic and relevant perspective.

Include healthcare assistants in the videos.

### PDSA cycle 2

In response to the feedback received from the care staff on areas of improvement, the video was refilmed incorporating the suggested modifications. This included a thorough review and redrafting of the script to personalise it for the care home staff and improving the font size of the text for improved readability. Moreover, following the suggestions provided, we also invited a member of the care staff to participate in the video, further enhancing its relevance and relatability.

The refilmed video was shown to 10 care staff members of another local care home. The pre-video survey demonstrated that four out of 10 participants had no knowledge and understanding of contractures (40%), five had basic knowledge and understanding of contractures (50%) and one had good knowledge and understanding of contractures (10%). When asked, how much you agree with the following statement: *I provide care for residents with contractures’*, seven reported, never’ (70%), two reported, often (20%) and one reported, always (10%).

When asked *if they ever used a tool or method to assess the risk of contractures for their residents*, all participants, reported, ‘no’ (100%) and all participants reported that they had never received any training related to contractures or postural management before (100%).

The post-video survey showed that 7 out of 10 participants reported good knowledge and understanding of contractures (70%), two reported basic knowledge and understanding of contractures (20%) and one reported extensive knowledge and understanding of contractures (10%). When asked, how much you agree with the following statement: ‘I provide care for residents with contractures’, six reported ‘often’ (60%), two reported ‘sometimes’ (20%) and two reported ‘always’ (20%).

The qualitative feedback received was positive and care staff suggested no further changes in the video.

### Dissemination

The refilmed video was disseminated via the official YouTube channel of NHS Dorset and is freely and easily accessible.^14^ The distribution of the video was further amplified through widespread sharing on social media platforms like Twitter/X. The video gained additional recognition by being featured in the frontline magazine of the Chartered Society of Physiotherapy.

## Lessons and limitations

### Lessons

The findings of this project suggest that a training based on an educational video has a positive impact on the knowledge and understanding regarding contractures among the care home staff. To our knowledge, this is the first QI project that developed and tested an educational approach specifically for care home staff.

In the first PDSA cycle, the video was developed and tested with the care home staff, and their feedback was incorporated into the second PDSA cycle, which involved refilming of the video. The refilmed video included a more personalised and relatable language for the care staff in the delivery of the training featuring a care staff member as a trainee. A key lesson learnt in this project was the importance of meaningful and active inclusion of the target group in the development process, which was missing from the initial video. This approach aligns with the principle of *Nothing about us, without us, which is* usually applied to patients but is equally relevant here. Allowing the care staff to see themselves in the video ensured that the content resonated with the target audience, potentially empowering them and improving their confidence in utilising appropriate measures to identify the risks associated with contractures and taking timely action.

Our results also indicate that a video-based intervention is an effective way to communicate and educate the care home staff about contractures. This has also been confirmed by the previous studies that video-based interventions have a positive impact towards encouraging a behavioural change.^15^

A duration of 9–10 min of video length was selected to deliver the most important topics relevant to contractures within a context of a care home while maintaining engagement and attention.

Moreover, the STOP acronym used as part of the ‘Let’s STOP contractures’ campaign in the educational video emerged as the most popular and easy to recall feature of the training. This acronym covers all important aspects of contracture prevention strategies that can be utilised in a care home. Research studies have demonstrated that the use of ‘to-be-remembered’ material such as mnemonic acronyms in the learning phase improves the learning and has a long-lasting effect on the retention of the educational material.^16^

The lessons learnt will be used to gradually widen the scope of this project and improvise and implement this project in other clinical settings, for example, hospitals and other target populations, informal carers or relatives who care for people with contractures or at risk of developing contractures.

### Limitations

The primary limitation of the project revolved around the small sample size across two sites, which hindered the possibility of conducting an advanced statistical analysis of the data; therefore, limiting the generalisability.

Another limitation was the pre–post-test design that did not include a control group and because specific elements of the video could not be isolated, it cannot be ascertained which parts of the video were the most important and easy to retain.

## Conclusion

This QI project demonstrated that the implementation of an educational video is a feasible and well-accepted way of increasing awareness and improving knowledge and understanding of contractures among care staff. It serves as a cost-effective, easy to retain, transferable and potentially sustainable way to address the lack or poor awareness of contractures among the care home staff. This can ultimately trigger early identification and prevention of contractures and improve the quality of care of the care home residents. Further research is required to identify long-term retention of educational information and evaluate the impact of other educational materials. Also, understanding how the new knowledge acquired by the care staff affects the quality of care provided to the residents and early identification and prevention of contractures.

## supplementary material

10.1136/bmjoq-2024-002923 online supplemental file 1

## Data Availability

All data relevant to the study are included in the article or uploaded as supplementary information.
