# Supplementary figures and images for: Development and evaluation of a quality improvement educational video on joint contractures for care home staff
Source: BMJ Open Qual. 2024 Dec 27;13(4):e002923. doi: 10.1136/bmjoq-2024-002923 (PMC11683971; doi:10.1136/bmjoq-2024-002923)

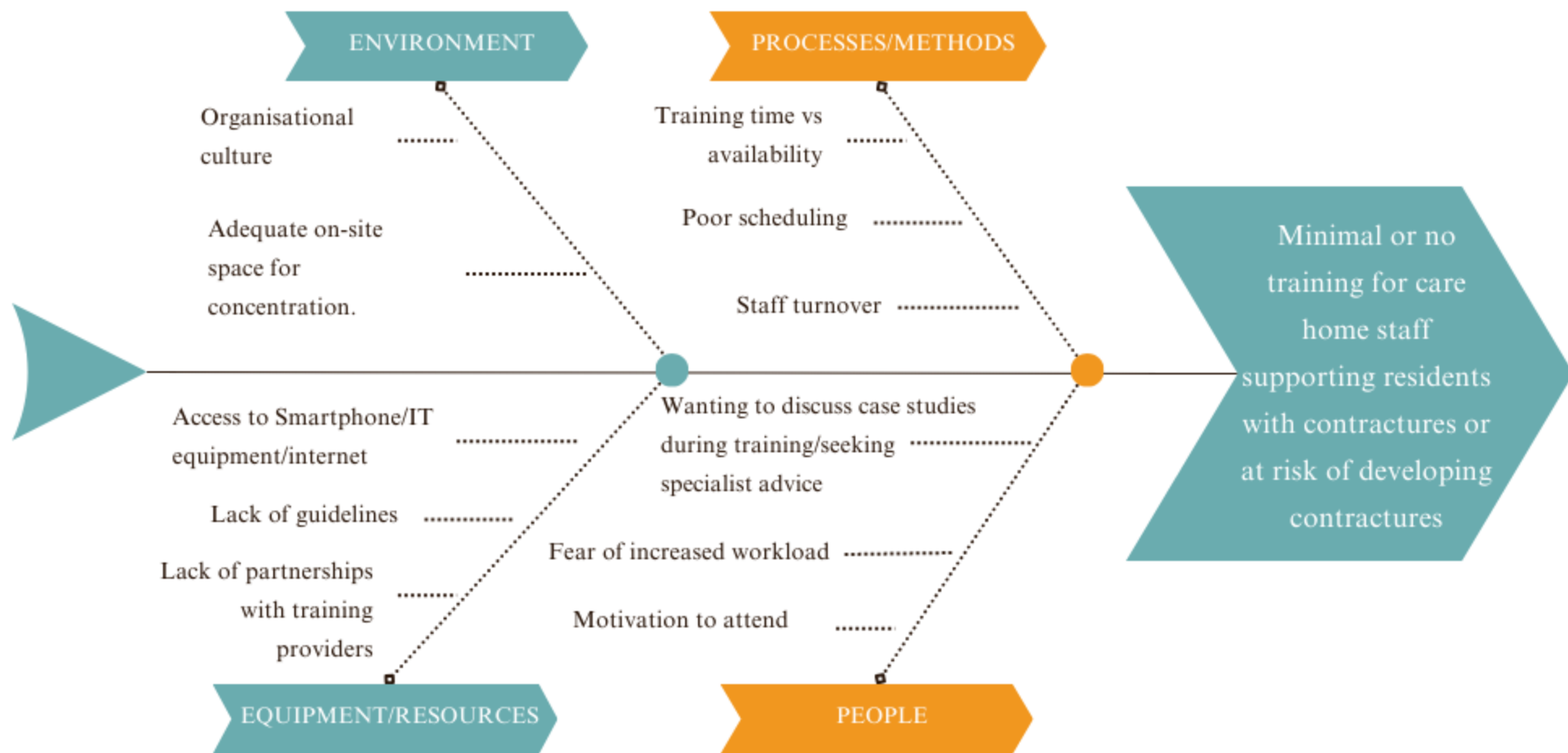

Supplement: online supplemental file 1 [file bmjoq-13-4-s001.pdf]
